# National record-linkage study of hospital admissions for schizophrenia in childhood and adolescence in England

**DOI:** 10.1007/s00787-021-01817-3

**Published:** 2021-06-18

**Authors:** Olena Seminog, Uy Hoang, Michael Goldacre, Anthony James

**Affiliations:** 1 Unit of Health-Care Epidemiology, Big Data Institute, Li Ka Shing Centre for Health Information and Discovery, Nuffield Department of Population Health, University of Oxford, Old Road Campus, Oxford, OX3 7LF UK; 2 Nuffield Department of Primary Health Care Sciences, University of Oxford, Oxford, UK; 3 Department of Psychiatry, University of Oxford, Oxford, UK

**Keywords:** Schizophrenia, Childhood onset, Children, Epidemiology, Electronic records

## Abstract

**Background:**

There is a lack of information on changes in hospital admission rates for childhood-onset schizophrenia (COS), or on patient characteristics, to inform clinical research and health service provision.

**Aims:**

To report age- and sex-specific incidence rates of hospital admissions and day patient care for schizophrenia (ICD-10 F20) and non-affective psychosis (ICD-10 F20-29), by year of occurrence and age, in childhood and adolescence.

**Methods:**

Population-based study using person-linked data for England (available 2001–2016); time-periods in single years and 4-year groups.

**Results:**

Hospitalised incidence for schizophrenia increased with increasing age, from 0.03 (95% confidence interval (CI) 0.02–0.05) and 0.01 (0–0.01) per 100,000 in, respectively, males and females aged 5–12 years, to 3.67 (3.44–3.91) in males and 1.58 (1.43–1.75) in females aged 13–17 years. There was no gender difference in hospitalised incidence rates in children aged 5–12, but in 13–17 years old, there was a male excess. Rates for schizophrenia were stable over time in 5–12 years old. In ages 13–17, rates for schizophrenia decreased between 2001–2004 and 2013–2016 in males, from 6.65 (6.04–7.31) down to 1.40 (1.13–1.73), and in females from 2.42 (2.05–2.83) to 1.18 (0.92–1.48). The hospitalisation rates for schizophrenia and non-affective psychosis, combined, in 13–17 years old decreased in males from 14.20 (13.30–15.14) in 2001–2004 to 10.77 (9.97–11.60) in 2013–2016, but increased in females from 7.49 (6.83–8.20) to 10.16 (9.38–11.00).

**Conclusions:**

The study confirms that childhood-onset schizophrenia is extremely rare, with only 32 cases identified over a 15-year period in the whole of England. The incidence of schizophrenia and non-affective psychosis increased substantially in adolescence; however, the marked reduction in the proportion of those diagnosed with schizophrenia in this age group suggests a possible change in diagnostic practice.

**Supplementary Information:**

The online version contains supplementary material available at 10.1007/s00787-021-01817-3.

## Introduction

Childhood-onset schizophrenia (COS), defined as onset before 13 years of age, is a serious, psychotic disorder with high rates of morbidity, mortality, and poor recovery [1, 2]—only 50% partially remit at 42-year follow-up [3]. In England, schizophrenia accounts for a quarter of all psychiatric admissions in the age group 10–18 years, and the admission rate increases with age [4]. It is generally thought that COS is very rare; however, epidemiological estimates of the incidence and prevalence of COS are sparse, which is a limitation given that these children require high levels of health resources due to the level of disabilities.

In this study, we describe the demographic characteristics and hospitalised incidence rates for COS and early-onset schizophrenia (EOS)—schizophrenia arising in adolescents between the ages of 13–17 years—alongside a broader category of non-affective psychosis for both age groups.

National datasets of inpatient electronic records are a valuable source of information on rare diseases. This study used linked national routine electronic patient records, an alternative methodology to the use of disease registries or cohort studies using personal follow-up of people with psychiatric conditions. We report on hospitalised incidence rates, covering inpatients and day-cases, for COS and EOS in England for the period 2001–2016 by sex. To set this in a wider historical context, we also consulted archived tables from the Oxford Record Linkage Study (ORLS) covering trends in psychiatric-service contacts for schizophrenia in the former Oxford NHS Region from 1966–1993 [5].

## Methods

### Datasets

#### Hospital episode statistics

We analysed a linked dataset of Hospital Episode Statistics (HES) for England from 2001 to 2016. The population of England was 55 million in 2016. In England, treatment in the National Health Service (NHS) hospitals is free. All hospital admissions and day-cases are included in the HES dataset and were called inpatients in this paper. The clinical diagnoses in HES are coded using the International Classification of Diseases (ICD). Each HES record contains information on the primary diagnosis, secondary diagnoses, demographic characteristics, and administrative variables such as dates of admission and discharge. Socio-economic status is measured using the patients’ area-level Index of Multiple Deprivation (IMD), which is a standard measure of neighbourhood deprivation recorded in HES. Ethnicity is self-reported in HES. Hospital records for every patient in the dataset were linked together in time-sequenced order of admissions. The record linkage was done centrally by NHS Digital.

#### Oxford Record Linkage Study (ORLS)

We also included historical data from a subset of the Oxford Record Linkage Study (ORLS), available as aggregated statistical tables held in the Unit of Health-Care Epidemiology (UHCE), University of Oxford. The ORLS is a regional dataset for the former Oxford NHS Region [5]. The ORLS data included outpatients (ambulatory care, provided by psychiatrists) from 1975 to 1993 as well as inpatients and day case care. Using these data enabled us to add historical context for the current study, and also acted as a sensitivity test for the national dataset of routine patient data, which is limited to hospital inpatient contacts only. The ORLS data and the national HES dataset were compiled independently of each other and, to that extent, each can be used to corroborate the findings in the other. The ORLS covered data collected from 1966. In addition, from 1975–1993, in the county of Oxfordshire, it included specialist psychiatric data collection on all ambulatory patients. The ORLS tabulations are available in quinquennial age groups (0–4, 5–9, 10–14 years). Therefore, we included children aged 5–14 years, as we were not able to restrict to under 13 years. The cumulative population denominators for the 44-year study period, expressed in person-years, were 699,900 in males and 664,100 in females.

#### Study design

The analyses in the national study included all children aged 5–17 years who had a hospital record with a diagnosis of schizophrenia and non-affective psychosis. We included people who were admitted to a hospital with schizophrenia recorded as the primary diagnosis, regardless of hospital specialty. This was done to achieve maximum coverage of all COS cases nationally. It is possible that some children, in particular, those with less severe presentations, might have been managed in a paediatric ward under the joint care of a paediatrician and a child psychiatrist.

#### Case identification

The cases of COS were defined using the ICD codes. In the national dataset, we used the ICD-10 codes F20 for schizophrenia and F20–29 for the broader ICD diagnostic block termed “schizophrenia, schizotypical and delusional disorders”. We refer to the latter block, in what follows, as non-affective psychosis. For the historical data from Oxfordshire, we used the corresponding diagnostic block codes from the earlier revisions of the ICD.

#### Consent statement

The datasets are anonymous; patients and contact details cannot be identified. This data-based study did not involve informed consent from participants.

## Data analysis

For the all-England study, we calculated age-specific incidence rates for schizophrenia in two age groups, 5–12 and 13–17 years. The rates were calculated for every calendar year between 2001 and 2016, which is the year range for which the datasets were available. Because of the small number of events in some age groups, to obtain more statistically robust results, we have also grouped years in four equal intervals, 2001–2004, 2005–2008, 2009–2012, and 2013–2016. The annual rates and rates for the broader time-periods were calculated by dividing the number of cases of schizophrenia at each age by the total number of children in England at that age. Age-specific population denominators were obtained from the Office for National Statistics.

In ORLS, the incidence rates were calculated by dividing the total number of cases of schizophrenia in the specified period of time by the average population in those years and in the relevant age, and by the number of years.

The rates were calculated for males and females separately and expressed per 100,000 age- and sex-specific population. The corresponding 95% confidence intervals were calculated assuming a Poisson distribution. We only included the first-ever record of diagnosed schizophrenia for each individual, which is the closest available measure of population incidence. If an individual had two or more admissions in one calendar year or in different calendar years, only the first one was included in the analysis.

We reported on the rates of previous hospital admissions for any psychiatric disorder in males and females with COS, recorded as either main or secondary diagnosis. We used the ICD-10 range of codes F00–F19 and F30–F99 to identify admissions for previous psychiatric conditions.

We calculated the proportion of people with COS diagnosed with autism, using the ICD-10 code F84 recorded in any position on the hospital discharge record.

## Ethical approval

The Central and South Bristol research ethics committee (No 04/Q2006/176) approved the building and analysis of the dataset of anonymised records for a multi-purpose programme of research by the Unit of Health-Care Epidemiology, University of Oxford.

## Results

### English national data

#### Study population characteristics

Over the course of 15 years, in a total all-England population of 55 million people, we identified 32 inpatient cases of childhood-onset schizophrenia in children aged 5–12 years, and 1,354 inpatient cases of early-onset schizophrenia in adolescents aged 13–17 years (Table 1). There was no gender difference in COS, but higher incidence rates of EOS in males than females (Table 1). More than 67% of males and 80% of females with COS were from the two lowest socio-economic quintiles as measured by the IMD. Ethnicity was recorded for 75% (n = 9 people) males and 87% (18) females with COS: of these, 33% (3) of males and 39% (7) of females were White, 22% (2) of males and 28% (5) of females were Black (Table 1). The distribution of cases by place of residence showed that 50% (6) of males and 25% (5) of females with COS lived in London (Table 1). Among COS patients, 50% (10) of females and none of the males had a recorded diagnosis of autism. Most children with COS, 83% (10) males and 80% (16) females, had a record of previous hospital admission for another psychiatric condition.

**Table 1:** Demographic characteristics of patients hospitalised with schizophrenia (F20) and non-affective psychosis (F20–29) from 2001 to 2016, by age group and sex

	F20				F20–F29			
	Males		Females		Males		Females	
Age group	5–12 years	13–17 years	5–12 years	13–17 years	5–12 years	13–17 years	5–12 years	13–17 years
	N (%)	N (%)	N (%)	N (%)	N (%)	N (%)	N (%)	N (%)
Total number (% of total)	12	960	20	394	117	2940	152	1979
Number of people in different socio-economic groups and % of total, by IMD quintiles								
Most deprived, Q1	5 (42%)	339 (37%)	8 (40%)	140 (37%)	38 (32%)	1023 (36%)	46 (31%)	676 (35%)
Q2	3 (25%)	227 (25%)	8 (40%)	98 (26%)	36 (31%)	681 (24%)	45 (30%)	420 (22%)
Q3	0	178 (19%)	2 (10%)	68 (17%)	18 (15%)	504 (18%)	25 (17%)	318 (17%)
Q4	2 (17%)	103 (11%)	2 (10%)	42 (11%)	12 (10%)	352 (12%)	21 (14%)	276 (14%)
Least deprived, Q5	2 (17%)	77 (8%)	28 (10%)	35 (9%)	13 (11%)	296 (10%)	13 (9%)	236 (12%)
Ethnicity								
White	3 (33%)	587 (70%)	7 (39%)	217 (62%)	59 (61%)	1675 (66%)	71 (56%)	1031 (67%)
Black	2 (22%)	112 (13%)	5 (28%)	67 (19%)	17 (18%)	369 (14%)	27 (21%)	253 (15%)
Asian	1 (11%)	67 (8%)	2 (11%)	29 (8%)	7 (7%)	214 (8%)	14 (11%)	132 (8%)
Other	3 (33%)	69 (8%)	4 (22%)	35 (10%)	14 (14%)	296 (12%)	15 (12%)	174 (10%)
Not recorded	3 (25%)	125 (13%)	2 (10%)	46 (12%)	20 (17%)	386 (13%)	47 (17%)	25 (16%)
Region of residence								
North East	0	60 (6%)	1 (5%)	19 (5%)	2 (2%)	139 (5%)	4 (3%)	62 (3%)
North West	2 (17%)	183 (19%)	4 (20%)	64 (16%)	21 (18%)	557 (19%)	27 (18%)	323 (16%)
Yorkshire and Humber	0	99 (10%)	0	29 (7%)	7 (6%)	224 (8%)	3 (2%)	139 (7%)
East Midlands	0	76 (8%)	3 (15%)	34 (8%)	4 (3%)	204 (7%)	13 (9%)	147 (7%)
West Midlands	1 (8%)	80 (8%)	3 (15%)	39 (10%)	12 (10%)	275 (9%)	16 (11%)	201 (10%)
East of England	0	58 (6%)	0	28 (7%)	10 (9%)	193 (7%)	12 (8%)	159 (8%)
London	6 (50%)	227 (24%)	5 (25%)	104 (26%)	44 (38%)	692 (24%)	41 (27%)	452 (23%)
South East	2 (17%)	75 (8%)	2 (10%)	40 (10%)	13 (11%)	335 (11%)	23 (15%)	296 (15%)
South West	1 (8%)	69 (7%)	2 (10%)	27 (7%)	4 (3%)	244 (8%)	11 (7%)	151 (8%)

#### Changes in incidence rates and age-specific rates of COS and EOS, 2001–2016

The number of cases of COS varied from year to year, with no cases in some years (Table 2 and Supplementary Tables 1 and 2). For males with COS, the incidence rates between 2001 and 2016 were very low, but stable as evident from the overlapping confidence intervals for rates of 0.03 (95%CI 0.01–0.05) per 100,000 in 2001 and 0.07 (0.01–0.27) in 2016, and for females 0.04 (0.00–0.23) in 2002 (no cases were recorded in 2001) and 0.04 (0.00–0.22) in 2016 (Table 2). After these small numbers were combined in equal time-periods to yield more power, no statistically significant changes in rates were observed (Table 3). Likewise, there was no statistically significant increase in rates of non-affective psychosis (F20-F29)—the confidence intervals overlapped for rates calculated in individual years, as well as time-periods: for males from 0.27 (0.11–0.56) in 2001 to 0.51 (0.28–0.86) in 2016 and for females from 0.24 (0.09–0.53) in 2001 to 0.73 (0.44–1.14) in 2016 (Table 2). When rates were calculated in time-periods, they were 0.32 (0.22–0.45) in 2001–2004 and 0.34 (0.24–0.48) in 2013–2016 in males and 0.32 (0.21–0.45) and 0.50 (0.37–0.66), respectively, in females (Table 3).

**Table 2:** Number of admitted cases of schizophrenia (F20) and non-affective psychosis (F20–F29) and hospitalised incidence in the first and last year of the study, and average estimates for a period 2001–2016, by age group and sex

Year	Population denominators	F20	Rates (95%CI) per 100,000	No. of cases	F20–F29
		N of cases			Rates (95%CI) per 100,000
Total N of cases and average rates					
2001	2,599,800	2	0.08 (0.01–0.29)	7	0.27 (0.11–0.56)
2016	2,722,500	2	0.08 (0.01–0.28)	14	0.51 (0.28–0.86)
Total N of cases and average rates					
2001–2016	40,557,900	12	0.03 (0.02–0.05)	117	0.29 (0.24–0.35)
Males, 13–17 years					
2001	1,615,200	114	7.06 (5.82–8.48)	226	13.99 (12.23–15.94)
2016	1,558,700	12	0.77 (0.40–4.34)	180	11.55 (9.92–13.36)
Total N of cases and average rates					
2001–2016	26,186,400	960	3.67 (3.44–3.91)	2940	11.23 (10.82–11.64)
Females, 5–12 years					
2001	2,475,000	0		6	0.24 (0.09–0.53)
2016	2,594,600	1	0.04 (0–0.22)	19	0.73 (0.44–1.14)
Total N of cases and average rates					
2001–2016	38,659,700	20	0.01 (0–0.01)	152	0.39 (0.33–0.46)
Females, 13–17 years					
2001	1,531,900	45	2.94 (2.14–3.93)	112	7.31 (6.02–8.80)
2016	1,480,500	17	1.15 (0.67–1.84)	152	10.27 (8.70–12.03)
Males, 5–12 years					
2001–2016	24,966,600	394	1.58 (1.43–1.74)	1979	7.93 (7.58–8.28)

**Table 3:** Time trends in hospitalised incidence rates for schizophrenia and non-affective psychosis presented in four time intervals, with the corresponding number of events and population denominators by age and sex

Year	Population denominators	N of cases	F20	No. of cases	F20–F29
			Rate (95%CI) per 100,000		Rate (95%CI) per 100,000
Males, 5–12 years					
2001–2004	10,300,000	7	0.07 (0.03–0.14)	33	0.32 (0.22–0.45)
2005–2008	9,936,400	1	0.01 (0–0.06)	20	0.20 (0.12–0.31)
2009–2012	9,812,900	1	0.01 (0–0.06)	28	0.29 (0.19–0.41)
2013–2016	10,483,300	3	0.03 (0.01–0.08)	36	0.34 (0.24–0.48)
Males, 13–17 years					
2001–2004	6,536,900	435	6.65 (6.04–7.31)	928	14.20 (13.30–15.14)
2005–2008	6,699,300	276	4.12 (3.65–4.64)	693	10.34 (9.59–11.14)
2009–2012	6,605,900	160	2.42 (2.06–2.83)	636	9.63 (8.89–10.41)
2013–2016	6,344,300	89	1.40 (1.13–1.73)	683	10.77 (9.97–11.60)
Females, 5–12 years					
2001–2004	9,833,900	5	0.05 (0.02–0.12)	31	0.32 (0.21–0.45)
2005–2008	9,469,700	5	0.05 (0.02–0.12)	30	0.32 (0.21–0.45)
2009–2012	9,361,900	5	0.05 (0.02–0.13)	41	0.44 (0.31–0.59)
2013–2016	9,994,200	5	0.05 (0.02–0.12)	50	0.50 (0.37–0.66)
Females, 13–17 years					
2001–2004	6,248,400	151	2.42 (2.05–2.83)	468	7.49 (6.83–8.20)
2005–2008	6,407,900	101	1.58 (1.28–1.92)	421	6.57 (5.96–7.23)
2009–2012	6,279,400	71	1.13 (0.88–1.43)	477	7.60 (6.93–8.31)
2013–2016	6,030,900	71	1.18 (0.92–1.48)	613	10.16 (9.38–11.00)

The incidence rates of schizophrenia were overall higher in adolescence, which reduced over the study period. In 2001, in males aged 13–17, the incidence rate for EOS (F20) was 7.06 (5.82–8.48), which decreased during the study period to 0.77 (0.40–4.34) in 2016 (Table 2). In females with EOS, the incidence rates were 2.94 (2.14–3.93) in 2001 and 1.15 (0.67–1.84) in 2016 (Table 2). Similarly, in time-period analyses, the rates in males decreased from 6.65 (6.04–7.31) in 2001–2004 to 1.40 (1.13–1.73) in 2013–2016, and in females from 2.42 (2.05–2.83) to 1.18 (0.92–1.48), respectively (Table 3). The pattern for non-affective psychosis (F20–F29) showed decreasing rates in males aged 13–17, and increasing rates in females in the same age group, as revealed by time-period analysis. In males, the rates declined from 14.20 (13.30–15.14) in 2001–2004 to 10.77 (9.97–11.60) in 2013–2016. In females, the rates were 7.49 (6.83–8.20) in 2001–2004 and 10.16 (9.38–11.00) in 2013–2016. (Table 3). The annual number of cases and hospitalised incidence rates for COS and EOS are provided as supplementary material (Supplementary Tables 1 and 2).

**Fig. 1:**
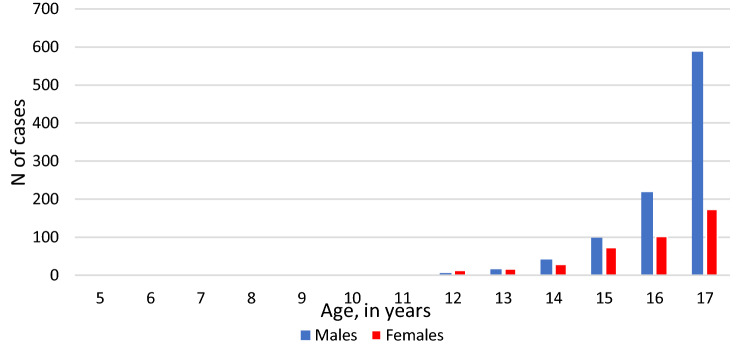
Age distribution of schizophrenia cases in individual years, 2001–2016, males and females

**Fig. 2:**
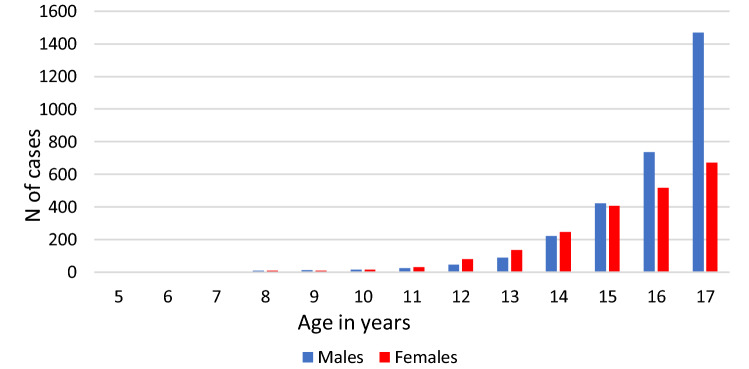
Age distribution of non-affective psychosis cases in individual years of age, 2001–2016, males and females

The number of schizophrenia cases increased with age (Fig. 1). For example, at ages 5, 6, and 7 years, there was only one case in females and no cases in males over the 15 years of observation. The great majority (90%) of schizophrenia cases were in the age group 14 and older. The male-to-female ratio changed with age: up to the age of 13 there were more cases in females than males (20 vs 12). The male excess of schizophrenia and non-affective psychosis started from the age of 14 years (Figs. 1 and 2).

#### COS incidence rates: Oxford Record Linkage Study

In the 44 years between 1966 and 2010, there were 28 males and 8 females aged 5–14 admitted to hospital for schizophrenia. The average annual rates expressed per 100,000 residents aged 5–14 were 1.66 in males and 0.49 in females.

In the 18 years between 1975 and 1993, when data collection on all specialist psychiatric contacts (outpatients as well as inpatients) was available, there were 12 males and 5 females with schizophrenia aged 5–14. The average annual rates expressed per 100,000 residents aged 5–14 were 1.70 in males and 0.73 in females.

## Discussion

### Summary of results

Childhood-onset schizophrenia is very rare indeed. There were only 32 hospitalised cases of COS in in the whole of England (population 55 million) over a period of 15 years, with a hospitalised incidence rate in 2016 in males of 0.03 per 100,000 population and in females of 0.01 per 100,000 population. There was no significant difference between the male and female rates of COS, whereas a male excess started from the age of 14 years. In line with other studies, we reported that a large proportion of children with schizophrenia were of a low socio-economic status [6, 7]. Half of females with COS were diagnosed with autistic spectrum disorder. There has been a striking reduction in the proportion of adolescents with psychosis (as judged by use of ICD codes F20–F29) receiving the diagnosis of schizophrenia (F20) during the period 2001–2016 (Supplementary Tables 1 and 2). The reason for this reduction in hospitalised incidence rates for adolescent males with schizophrenia is not entirely clear, although judging from the timing, with the main decline occurring in the early years of the study, the introduction of the early intervention services (EIS) in 1999, with the aim of reducing admissions, could be a factor. EIS is a dedicated support service with an explicit aim to treat cases in the community, rather than hospitals with attendant problems of inpatient care in some cases. We have no explanation of why this should affect males more than females.

#### Comparison with other studies

Our estimates of the incidence rates of schizophrenia in children are similar to the rates reported in the literature, albeit that the literature is fairly sparse. Early studies of COS, in North Dakota in 1987, found a low prevalence rate of 1.9 per 100,000 which is consistent with the incidence rates estimated using the ORLS data [8]. In three large districts in Germany, Hafner found that admissions for schizophrenia before age 12 hardly ever occurred [9]. Similarly, this study reported only 32 cases in one and a half decades in the whole of England. Likewise, a nationwide study from Denmark, with a population around 5 million, showed COS to be very rare with 4 admissions before 13 years and 28 before 15 years of age between 1970 and 1993 [10]. A survey of UK and Irish child and adolescent psychiatrists reported 8 cases of schizophrenia below the age of 14, and estimated a low incidence rate in the range between 0.21 and 1 per 100,000 in children aged 10–14 [11].

Our results confirm the very low rates of admission for COS with an increase in schizophrenia admissions during adolescence. Retrospective surveys indicate that less than 1% of all schizophrenic disorders manifest themselves before age 10, and 4% before age 15 [12]. The relation between COS and autism has been described in the literature, with reports of 30–50% of COS patients having autism [13], similar to the 50% among females reported here. As yet, there have been no studies indicating regional variations in the incidence of COS, as is well documented for schizophrenia in older age groups [14]. Further research on geographical variation in COS is needed.

An overall higher rate of schizophrenia in males has been observed [15], although there are fewer gender differences reported in COS [16, 17]. However, male patients were reported to develop COS at younger ages than females [15, 16]. A Danish register study reported on long-term trends in schizophrenia incidence in people under 18 years, and found that over the period 1971–2010, the incidence rates declined in males and increased in females [17]. These findings are similar to our findings on the reduction in EOS incidence in males. A later Danish study confirmed the excess of schizophrenia spectrum disorders in adolescent females versus males, with rates at 0.76% (95% CI, 0.72%-0.80%) vs 0.48% (95% CI, 0.39%-0.59%), respectively [18]. We found more cases of COS in females than in males, but no significant difference in incidence rates. There were no details of pubertal status; however, this switch to a male predominance occurs over the normal pubertal age.

In this study, we found between 22–28% of those with COS and 13–19% with EOS were Black (Table 1); according to the most recent census 3.3% of the total population of England and Wales are Black [19], suggesting a higher proportion of Black minorities in those with schizophrenia. An excess of schizophrenia cases in Black minorities appears common to all age groups, with similar findings reported in adults [20].

#### Strengths and limitations

Using a large national dataset of electronic patient records to study schizophrenia in children, we identified the largest number of cases of COS to date. It is clear that with such low incidence rates, general population surveys might have a limited value. Early studies were hampered by not only the varied presentation of COS, but variable diagnostic criteria [21]. Clinical surveys have limitations—notably, sampling bias and non-standardised diagnosis. Indeed, recent work suggests that not using standardised interviews leads to an underestimate of the rates of early-onset schizophrenia, especially in cases with intellectual disability and neurodevelopmental disorders [22]. The validity of Hospital Episode Statistics for identifying cases of schizophrenia in adults is high, with positive predictive value of 90% [23]. To date, however, there are no similar validation studies for schizophrenia in children.

In the historical ORLS dataset, the psychiatric-service counts were only available in standard 5-year age groups. We had to report the number of events and incidence rates for schizophrenia for the age group 5–14 years.

The hospital data are likely to underestimate the true incidence of psychiatric conditions in the population. However, schizophrenia under 13 years is a severe disorder, often presenting with florid psychopathology, making it more likely that children with schizophrenia are admitted for investigation and treatment. Thus, the rates of admission for COS are likely to approximate the true population incidence rates. The methodology does not allow an assessment of age of true onset, so it is possible that some COS cases could have been first identified as admissions in adolescence or early adulthood. Significant national variation in COS geography is indicated by the fact that nearly one-third of cases of COS in this study came from London, which is disproportionate to its population size, comprising about 15% of the English population of 5 to 12 year olds (the total average population of England in the corresponding age group in 2001–2016 was 4,951,100 vs London 760,078) and 14% of the 13 to 17 (3,197,063 vs 450,346, respectively). Even allowing for higher rates of childhood psychosis seen in urban settings [24], this suggests possible differential use of health services or variable diagnostic practises geographically.

In considering the decline in admissions for schizophrenia in adolescent males, we note that there has been no change in the diagnostic system, ICD-10 in use throughout. It is possible that psychiatrists may have become more reluctant to make the specific diagnosis of schizophrenia in the more recent years. Making a diagnosis of COS is complex: psychotic symptoms in childhood are relatively common. UK Data from the Avon Longitudinal Study of Parents and Children (ALSPAC) birth cohort reveal a 6-month period prevalence of narrow psychosis-like symptoms in 11 years old to be 5.6% (95% CI 5.1–6.2) [25]. Furthermore, these symptoms are not specific for schizophrenia [26]. Making the diagnosis, therefore, requires expertise to avoid the high rate of false positives involving transient psychotic states [27]. However, the diagnosis of schizophrenia in children and adolescence, once made, is stable [28].

Psychiatrists are likely to face considerable difficulties in making a diagnosis of schizophrenia in this age group because of uncertainties, including considerable symptom overlap with pervasive developmental disorders, especially in males [16]; stigma, and negative connotation and perceived lack of diagnostic validation attributed to the diagnosis of schizophrenia [29]. Indeed, there is evidence from early intervention services of a very long delay of making the diagnosis of first episode psychosis in adolescence [30]. All these factors point to a possible failure to make a diagnosis of COS, and under-reporting, which could contribute to the generally low reported rates of COS. We reported that about 80% of COS patients had a previous hospital admission for other psychiatric conditions prior to the first record of schizophrenia, which is consistent with the findings from a Danish national study, showing an increased risk of schizophrenia following any previous psychiatric admission [31].

An alternative explanation for the low rate of admissions for COS could be that prodromal and comorbid cases are diagnosed and admitted for different reasons. There is a recognised link between childhood psychiatric disorders—especially opposition defiant disorder, anxiety, and attention deficit hyperactivity disorder—and the later development of schizophrenia [31]. A further caution is the diagnostic issue arising from the relationship of autistic spectrum disorders to non-affective psychosis [13].

In England, the proportion of beds in the private or independent sector is increasing. This suggests that our figures for EOS are likely to be an underestimate; however, this does not apply to COS, as in the UK, the private sector does not provide psychiatric inpatient care for children (32).

In conclusion, this is the largest epidemiological study of schizophrenia diagnosed before the age of 13 years. Our findings confirm that childhood-onset schizophrenia is rare, but with age, it rises to become one of the most common and costly psychiatric disorders in adulthood. These figures may help guide the necessary care and management provisions for this serious mental disorder, as well as inform and stimulate further research into this condition.

## Supplementary Information

Below is the link to the electronic supplementary material.

Supplementary file1 (DOCX 18 KB)

## Data Availability

Raw data were provided by NHS Digital. Derived data supporting the findings of this study are available from the corresponding author OS upon reasonable request.

## Code availability

Analysis was done using the SAS Management Studio code developed by the UHCE IT team, and not available as an open source code.
