# Supplementary material for: National record-linkage study of hospital admissions for schizophrenia in childhood and adolescence in England
Source: Eur Child Adolesc Psychiatry. 2021 Jun 18;31(12):1943–51. doi: 10.1007/s00787-021-01817-3 (PMC9663394; doi:10.1007/s00787-021-01817-3)
Supplement: Supplementary file 1 — Supplementary file1 (DOCX 18 KB) [file 787_2021_1817_MOESM1_ESM.docx]

Supplemental Materials.

Supplement. Table 1. Number of cases, hospitalised incidence rates of schizophrenia and all non-affective psychoses by calendar year, proportion of cases specified as schizophrenia among all non-affective psychosis, in males and females aged 5 to 12 years

|  | Male | | | | | |  | Female | | | | | |
| --- | --- | --- | --- | --- | --- | --- | --- | --- | --- | --- | --- | --- | --- |
| Year | Population denominators | N of events | Rates  /100,000 (95%CI) | N of events | Rates/100  0000 (95%CI) | % of schizophrenia among all non-affective psychosis |  | Population denominators | N of events | Rates  /100,000 (95%CI) | N of events | Rates  /100,000 (95%CI) | % of schizophrenia among all non-affective psychosis |
| F20 | | | | F20-29 | | |  | F20 | | | F20-F29 | | |
| 2001 | 2599800 | 2 | 0.08 (0.01-0.28) | 7 | 0.27 (0.11-0.56) | 29 |  | 2475000 | 0 | 0 | 6 | 0.24 (0.09-0.53) | 0 |
| 2002 | 2593800 | 4 | 0.15 (0.04-0.40) | 12 | 0.46 (0.24-0.81) | 33 |  | 2469500 | 1 | 0.04 (0.00-0.23) | 8 | 0.32 (0.14-0.64) | 13 |
| 2003 | 2579200 | 1 | 0.04 (0.00-0.22) | 5 | 0.19 (0.06-0.45) | 20 |  | 2457900 | 2 | 0.08 (0.01-0.29) | 8 | 0.33 (0.14-0.64) | 25 |
| 2004 | 2552500 | 0 |  | 9 | 0.35 (0.16-0.67) | 0 |  | 2431500 | 2 | 0.08 (0.01-0.30) | 9 | 0.37 (0.17-0.70) | 22 |
| 2005 | 2524100 | 1 | 0.04 (0.00-0.22) | 6 | 0.24 (0.09-0.52) | 17 |  | 2400800 | 0 | 0 | 3 | 0.13 (0.03-0.37) | 0 |
| 2006 | 2494600 | 0 |  | 2 | 0.08 (0.01-0.29) | 0 |  | 2377100 | 2 | 0.08 (0.01-0.30) | 10 | 0.42 (0.20-0.77) | 20 |
| 2007 | 2466500 | 0 |  | 7 | 0.28 (0.11-0.59) | 0 |  | 2353700 | 1 | 0.04 (0.00-0.24) | 8 | 0.34 (0.15-0.67) | 13 |
| 2008 | 2451200 | 0 |  | 5 | 0.20 (0.07-0.48) | 0 |  | 2338100 | 2 | 0.09 (0.01-0.31) | 9 | 0.39 (0.18-0.73) | 22 |
| 2009 | 2444200 | 0 |  | 8 | 0.33 (0.14-0.65) | 0 |  | 2331500 | 1 | 0.04 (0.00-0.24) | 11 | 0.47 (0.24-0.84) | 9 |
| 2010 | 2438400 | 0 |  | 5 | 0.21 (0.07-0.48) | 0 |  | 2328200 | 2 | 0.09 (0.01-0.31) | 15 | 0.64 (0.36-1.06) | 13 |
| 2011 | 2451500 | 1 | 0.04 (0.00-0.23) | 12 | 0.49 (0.25-0.86) | 8 |  | 2338100 | 2 | 0.09 (0.01-0.31) | 10 | 0.43 (0.21-0.79) | 20 |
| 2012 | 2478800 | 0 | 0.00 (0.00-0.00) | 3 | 0.12 (0.03-0.35) | 0 |  | 2364100 | 0 | 0 | 5 | 0.21 (0.07-0.49) | 0 |
| 2013 | 2526700 | 1 | 0.04 (0.00-0.22) | 8 | 0.32 (0.14-0.62) | 13 |  | 2409700 | 2 | 0.08 (0.01-0.30) | 12 | 0.50 (0.26-0.87) | 17 |
| 2014 | 2582500 | 0 |  | 8 | 0.31 (0.13-0.61) | 0 |  | 2462100 | 1 | 0.04 (0.00-0.23) | 9 | 0.37 (0.17-0.69) | 11 |
| 2015 | 2651600 | 0 |  | 6 | 0.23 (0.08-0.49) | 0 |  | 2527800 | 1 | 0.04 (0.00-0.22) | 10 | 0.40 (0.19-0.73) | 10 |
| 2016 | 2722500 | 2 | 0.07 (0.01-0.27) | 14 | 0.51 (0.28-0.86) | 14 |  | 2594600 | 1 | 0.04 (0.00-0.22) | 19 | 0.73 (0.44-1.14) | 5 |

Supplement. Table 2. Number of cases, hospitalised incidence rates of schizophrenia and all non-affective psychoses by calendar year, proportion of cases specified as schizophrenia among all non-affective psychosis, in males and females aged 13 to 17 years

| Males | | | | | | |  | Females | | | | | |
| --- | --- | --- | --- | --- | --- | --- | --- | --- | --- | --- | --- | --- | --- |
| F20 | | | | F20-F29 | | |  | F20 | | | F20-F29 | | |
| Year | Population denominators | N of events | Rates  /100,000 (95%CI) | N of events | Rates  /100,000 (95%CI) | % of schizophrenia among all non-affective psychosis |  | Population denominators | N of events | Rates  /100,000 (95%CI) | N of events | Rates  /100,000 (95%CI) | % of schizophrenia among all non-affective psychosis |
| 2001 | 1615200 | 114 | 7.06 (5.82-8.48) | 226 | 13.99 (12.23-15.94) | 50 |  | 1531900 | 45 | 2.94 (2.14-3.93) | 112 | 7.31 (6.02-8.80) | 40 |
| 2002 | 1629100 | 116 | 7.12 (5.88-8.54) | 223 | 13.69 (11.95-15.61) | 52 |  | 1551500 | 37 | 2.38 (1.68-3.29) | 112 | 7.22 (5.94-8.69) | 33 |
| 2003 | 1637900 | 101 | 6.17 (5.02-0.49) | 254 | 15.51 (13.66-17.54) | 40 |  | 1571700 | 31 | 1.97 (1.34-2.80) | 116 | 7.38 (6.10-8.85) | 27 |
| 2004 | 1654700 | 104 | 6.29 (5.14-1.62) | 225 | 13.60 (11.88-15.50) | 46 |  | 1593300 | 38 | 2.38 (1.69-3.27) | 128 | 8.03 (6.70-9.55) | 30 |
| 2005 | 1671300 | 100 | 5.98 (4.87-8.28) | 217 | 12.98 (11.31-14.83) | 46 |  | 1607300 | 33 | 2.05 (1.41-2.88) | 117 | 7.28 (6.02-8.72) | 28 |
| 2006 | 1675200 | 64 | 3.82 (2.94-9.88) | 171 | 10.21 (8.74-11.86) | 37 |  | 1599600 | 25 | 1.56 (1.01-2.31) | 98 | 6.13 (4.97-7.47) | 26 |
| 2007 | 1681400 | 47 | 2.80 (2.05-0.72) | 132 | 7.85 (6.57-9.31) | 36 |  | 1602200 | 21 | 1.31 (0.81-2.00) | 117 | 7.30 (6.04-8.75) | 18 |
| 2008 | 1671400 | 65 | 3.89 (3.00-0.96) | 173 | 10.35 (8.87-12.01) | 38 |  | 1598800 | 22 | 1.38 (0.86-2.08) | 89 | 5.57 (4.47-6.85) | 25 |
| 2009 | 1659000 | 33 | 1.99 (1.37-3.79) | 142 | 8.56 (7.21-10.09) | 23 |  | 1584700 | 25 | 1.58 (1.02-2.33) | 106 | 6.69 (5.48-8.09) | 24 |
| 2010 | 1657900 | 48 | 2.90 (2.13-1.84) | 170 | 10.25 (8.77-11.92) | 28 |  | 1574400 | 20 | 1.27 (0.78-1.96) | 111 | 7.05 (5.80-8.49) | 18 |
| 2011 | 1653700 | 36 | 2.18 (1.52-5.01) | 154 | 9.31 (7.90-10.90) | 23 |  | 1568600 | 12 | 0.77 (0.40-1.34) | 120 | 7.65 (6.34-9.15) | 10 |
| 2012 | 1635300 | 43 | 2.63 (1.90-9.54) | 170 | 10.40 (8.89-12.08) | 25 |  | 1551700 | 14 | 0.90 (0.49-1.51) | 140 | 9.02 (7.59-10.65) | 10 |
| 2013 | 1619300 | 36 | 2.22 (1.56-5.08) | 154 | 9.51 (8.07-11.14) | 23 |  | 1536700 | 20 | 1.30 (0.80-2.01) | 146 | 9.50 (8.02-11.17) | 14 |
| 2014 | 1596400 | 21 | 1.32 (0.81-8.01) | 159 | 9.96 (8.47-11.63) | 13 |  | 1519900 | 18 | 1.18 (0.70-1.87) | 152 | 10.00 (8.47-11.72) | 12 |
| 2015 | 1569900 | 20 | 1.27 (0.78-7.97) | 190 | 12.10 (10.44-13.95) | 11 |  | 1493800 | 16 | 1.07 (0.61-1.74) | 163 | 10.91 (9.30-12.72) | 10 |
| 2016 | 1558700 | 12 | 0.77 (0.40-4.34) | 180 | 11.55 (9.92-13.36) | 7 |  | 1480500 | 17 | 1.15 (0.67-1.84) | 152 | 10.27 (8.70-12.03) | 11 |
